# Sex Modulates the Influence of APOE Genotype on Sleep and Sleep‐Dependent Memory Consolidation in Older Adults

**DOI:** 10.1002/alz.090492

**Published:** 2025-01-03

**Authors:** Negin Sattari, Bryce A Mander, Abhishek Dave, Kitty K. Lui, Kate E Sprecher, Miranda G. Chappel‐Farley, Brady A Riedner, Barbara B. Bendlin, Ruth M Benca

**Affiliations:** ^1^ Department of Psychiatry and Human Behavior, University of California, Irvine, Irvine, CA USA; ^2^ University of California, Irvine, Irvine, CA USA; ^3^ Department of Cognitive Sciences, University of California, Irvine, Irvine, CA USA; ^4^ SDSU / UC San Diego Joint Doctoral Program in Clinical Psychology, San Diego, CA USA; ^5^ University of Wisconsin, Madison, WI USA; ^6^ University of Wisconsin ‐ Madison, Madison, WI USA; ^7^ University of Wisconsin‐Madison, Madison, WI USA; ^8^ Wake Forest School of Medicine, Wake Forest University, Winston‐Salem, NC USA

## Abstract

**Background:**

Alzheimer’s disease (AD) is a world‐wide healthcare crisis among older adults. Sex, aging, and apolipoprotein E (*APOE*) genotype are among the most impactful risk factors for AD. Sleep is beneficial for memory and changes with age. We aimed to test whether sex and *APOE* (ε4‐carriers/non‐carriers) interact to impact sleep‐dependent memory consolidation (SDMC).

**Methods:**

We tested 67 older adults (41 women, 25 ε4‐carriers, mean±SD; 61.8±6.1 years). Participants encoded word‐pairs in the evening, were tested immediately, then underwent overnight in‐lab polysomnography, and performed a delayed test in the morning. SDMC was computed as a difference score (morning‐evening). Time spent in non‐rapid eye movement (NREM) sleep stages, and electroencephalography (EEG) spectral power in frontal canonical frequency bands during NREM sleep, were quantified. ANOVA, independent t‐tests, and bivariate correlation analyses were employed in the study.

**Results:**

A sex × APOE interaction predicted SDMC (p = 0.02). Post hoc analysis revealed no difference between male ε4‐carriers and non‐carriers (p = 0.36), but among women, ε4‐non‐carriers exhibited increased deterioration (more forgetting) compared to ε4‐carriers (p = 0.02). Additionally, male ε4‐carriers had worse memory retention than female ε4‐carriers (p = 0.01). Further analysis showed that female ε4‐carriers spent more time in N3 sleep (p = 0.01), correlating with better SDMC (r = 0.72, p = 0.02), while male ε4‐carriers spent more time in N2 sleep (p = 0.04), also linked to better SDMC (r = 0.68, p = 0.03). Fisher r‐to‐z transformation indicated significant differences in these associations (p = 0.01). Regardless of sex, among ε4 non‐carriers, no significant associations were found between theta, alpha, slow‐sigma, fast‐sigma during NREM sleep and memory (all ps>0.17). Among ε4‐carriers, male participants showed no significant associations (all ps>0.54), whereas females exhibited positive associations between memory and theta (r = 0.80, p = 0.005), alpha (r = 0.82, p = 0.004), slow‐sigma (r = 0.85, p = 0.002), and fast‐sigma (r = 0.77, p = 0.009).

**Conclusion:**

Our study reveals that the interplay of sex and APOE affects sleep patterns and memory consolidation in older adults. Notably, distinct non‐rapid eye movement (NREM) sleep stages impact Slow Delta and Theta Modulation (SDMC) differently in male and female APOE ε4‐carriers. While enhanced sleep quality may mitigate APOE and sex‐related cognitive decline in later life, considering sex‐related differences in memory benefits from sleep is crucial for older adults at high risk for AD.

